# Practice list size, workforce composition and performance in English general practice: a latent profile analysis

**DOI:** 10.1186/s12875-024-02462-w

**Published:** 2024-06-11

**Authors:** Alfred Bornwell Kayira, Helena Painter, Rohini Mathur, John Ford

**Affiliations:** Wolfson Institute of Population Health, Barts and The London School of Medicine and Dentistry, Queen Mary University of London, 4 Newark Street, Whitechapel, London, UK

**Keywords:** General practice, Practice performance, Primary care, England, Latent profile analysis

## Abstract

**Background:**

Following government calls for General Practices in England to work at scale, some practices have grown in size from traditionally small, General Practitioner (GP)-led organisations to large multidisciplinary enterprises. We assessed the effect of practice list size and workforce composition on practice performance in clinical outcomes and patient experience.

**Methods:**

We linked five practice-level datasets in England to obtain a single dataset of practice workforce, list size, proportion of registered patients ≥ 65 years of age, female-male sex ratio, deprivation, rurality, GP contract type, patient experience of care, and Quality and Outcomes Framework (QOF) and non-QOF clinical processes and outcomes. Latent Profile Analysis (LPA) was used to cluster general practices into groups based on practice list size and workforce composition. Bayesian Information Criterion, Akaike Information Criterion and deliberation within the research team were used to determine the most informative number of groups. One-way ANOVA was used to assess how groups differed on indicator variables and other variables of interest. Linear regression was used to assess the association between practice group and practice performance.

**Results:**

A total of 6024 practices were available for class assignment. We determined that a 3-class grouping provided the most meaningful interpretation; 4494 (74.6%) were classified as ‘Small GP-reliant practices’, 1400 (23.2%) were labelled ‘Medium-size GP-led practices with a multidisciplinary team (MDT) input’ and 131 (2.2%) practices were named ‘Large multidisciplinary practices’. Small GP-reliant practices outperformed larger multidisciplinary practices on all patient-reported indicators except on confidence and trust where medium-size GP-led practices with MDT input appeared to do better. There was no difference in performance between small GP-reliant practices and larger multidisciplinary practices on QOF incentivised indicators except on asthma reviews where medium-size GP-led practices with MDT input performed worse than smaller GP-reliant practices and immunisation coverage where the same group performed better than smaller GP-reliant practices. For non-incentivised indicators, larger multidisciplinary practices had higher cancer detection rates than small GP-reliant practices.

**Conclusion:**

Small GP-reliant practices were found to provide better patient reported access, continuity of care, experience and satisfaction with care. Larger multidisciplinary practices appeared to have better cancer detection rates but had no effect on other clinical processes and outcomes. As England moves towards larger multidisciplinary practices efforts should be made to preserve good patient experience.

## Background

In most countries general practice is facing unprecedented demand, exacerbating a workforce crisis [1]. In England, between July 2022 and June 2023, an average of 1.35 million appointments were booked per day, 43% of these took place on the same day and nearly half (47.4%) were delivered by a General Practitioner (GP) [2]. While demand for healthcare is increasing, fewer GPs are joining the profession in the UK than the number leaving or retiring [2]. As of June 2023, there were 2,212 fewer fully qualified FTE GPs compared to September 2015, 18.8% of whom were lost in the preceding 12 months [2].

To address these challenges, policy makers in Egland have been encouraging general practices to work at scale (i.e., work together to deliver services to larger populations) [3, 4]. Between 2014 and 2018 the policy guidance was that practices should merge and form larger business entities [3]. After 2018, practices were encouraged to adopt a federated model of working by forming groups linked by different types of agreements while retaining variable degrees of autonomy [4]. This culminated in the creation of Primary Care Networks (PCNs) in 2019 [5]. Under a PCN, groups of practices work closely together and with other services such as mental health, social care, pharmacy and community services to provide care to people in their local areas [5]. Working at scale is intended to help practices become more efficient and sustainable through sharing resources and expertise [6, 7]. These policies have led to a 20% decrease in general practices in England, from 8,106 in April 2013 to 6,495 in June 2022, due to mergers or closures [8].

Empirical research on the impact of growth in practice size has consistently yielded mixed results. Larger practices tend to score higher in financially incentivised Quality and Outcomes Framework (QOF) and other clinical and preventive care indicators such as fewer emergency hospital admissions for ambulatory care sensitive conditions, timely referral of patients to secondary care and independent sector providers, use of investigations and clinical guidelines, vaccination rates and cervical cancer screening [9–11]. Smaller practices generally have better performance in patient experience indicators such as access, continuity of care and overall satisfaction [9, 12, 13]. However, there are smaller practices which do well in clinical indicators as there are large practices which report good patient experience [11, 14, 15].

The impact of workforce composition on outcomes is also variable and likely to relate to different skillsets and roles of different practitioners [16].

Much of the existing research has examined practice size either in terms of absolute list size, list size per GP or as single-handed versus group practices [9, 17]. However, as staff teams become more multidisciplinary the composition of different roles in the practice is becoming increasingly important. We hypothesised that there exists distinct patterns of practice list size and workforce composition which may be associated with practice performance. We sought to identify these practice profiles or subgroups and assess whether membership to a particular group determined how a GP practice performed in primary care indicators.

## Methods

We used Latent Profile Analysis, a finite mixture modelling method that seeks to identify unobserved subpopulations from one super population [18, 19], to identify latent practice profiles or subgroups, and assessed whether membership to a particular group was associated with practice performance in clinical and nonclinical indicators.

### Datasets and data linkage

This cross-sectional study involved linking five datasets (General Practice workforce, General Practice Patient Survey, NHS Payments to General Practice, QOF and National General Practice Profiles) using practice code to create one dataset of practice workforce, list size, percentage of registered patients that are 65 years of age or older, general practice index of multiple deprivation (IMD), rurality and General Practice performance indicators.

We used the General Practice workforce data, as of 31 January 2023, to provide information on general practice workforce. General practice workforce data is available from NHS Digital [20]. We used Full-time equivalent (FTE) data for four staff groups (GPs, Nurses, DPC and administrative staff), with breakdowns of individual job roles within these high-level groupings. 1FTE is equivalent to 37.5 work hours a week. The workforce dataset also contains information on practice list size, sex (proportion male/female) and age of registered patients.

We used the 2022 General Practice Patient Survey (GPPS) [21] to provide information on patient reported indicators including access, continuity of care, confidence and trust in healthcare professionals, patient experience of and satisfaction with care. The GPPS in an online questionnaire sent yearly to randomly selected individuals registered with general practices in England.

We used 2021/22 Quality and Outcomes Framework (QOF) datasets. These are financial incentives linked to pre-specified quality targets for practices in the UK [22].

We used the National General Practice Profiles data sets, accessible from Office for Health Improvement and Disparities [23] to provide data on non-incentivised (non-QOF) clinical/public health indicators and practice-level socio-economic deprivation as measured by practice’s Index of Multiple Deprivation.

We extracted rurality data (classify practices as rural or urban) and GP contract type (different packages of services that GP practices provide based on local population needs) from NHS payments to general practice datasets (2021/2022) [24].

**Fig. 1:**
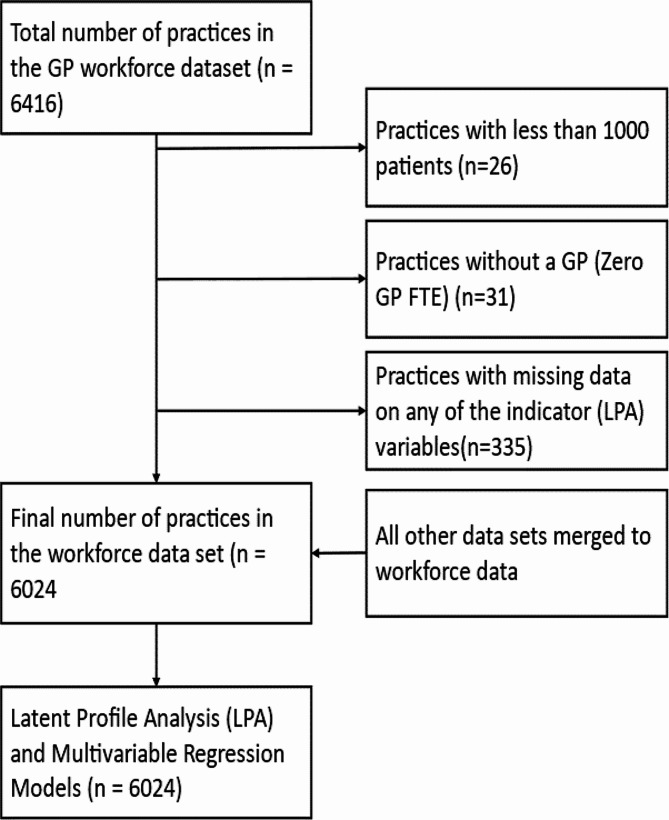
Data merging process and exclusion criteria

The workforce dataset served as the primary dataset to which all other datasets were merged. Figure 1 shows the data merging process and exclusion criteria. Practices with < 1000 registered patients and those without a GP were excluded because these are atypical practices, and are not included in some general practice profiles [23].

### Latent profile analysis

Latent Profile Analysis (LPA) was used to group practices according to practice list size and workforce composition. We included GPs (doctors), nurses, paramedics, pharmacist, health care assistant (HCA), administrative staff and other allied health professionals (AHP) (Table 1). Practitioners were grouped together where roles are sufficiently similar.

**Table 1:** Variables used to generate practice profiles/groups in LPA

Variable/Practitioner type	Description
Practice size	Number of people registered at a general practice
General Practitioner (GP)	All GPs (fully qualified permanent GPs, training grade and locum GPs)
Nurse specialist	Advanced nurse practitioners, nurse specialist and nurse partner
Nurse generalist	All other nurses doing clinical roles (except apprenticeship nurses)
Pharmacist	Pharmacy advanced practitioner, Pharmacist, Pharmacy technician and Dispenser
Health Care Assistant (HCA)	Health care assistants and nurse associates
Administration	Includes Administration manager, Administration management partner, Medical secretary and Receptionists
Allied Healthcare Professionals (AHP)	Includes Advanced Paramedic Practitioner, Advanced Physiotherapy Practitioner, Advanced Podiatry Practitioner, Advanced Occupational Therapy Practitioner, Dietician, Physiotherapist, Podiatrists, Physician associate, Occupational therapists, Paramedics, Apprentice physiotherapists

We explored LPA that generated 2 to 5 groups. Model goodness of fit, as measured by Bayesian Information Criterion (BIC) and Akaike Information Criterion (AIC), entropy (a measure of how distinct the derived groups are), and interpretability of the derived groups and how they might be applied in practice [19, 25] were used to determine the most informative number of groups. Through LPA, each general practice was allocated membership to one group for which they had the highest probability. We considered a class membership probability of < 50% as unacceptable (i.e., an indication of considerable uncertainty in class membership) [18, 19] and class membership probability of ≥ 80% as desirable [26]. Descriptors for each group were determined through discussion within the research team after examining how the groups differed on indicators variables. LPA was undertaken in R studio using the tidyLPA package.

Group means were calculated for continuous variables and frequencies were used to summarise categorical variables. One-way ANOVA (Analysis of Variance) was used to compare how the derived practice groups differed on indicator variables and on other practice characteristics. A Chi square or Fisher’s exact test (as appropriate) was used for categorical practice characteristics. One-way ANOVA was also used to assess how the derived practice groups compared on performance indicators. An alpha level of 0.05 was used in both instances.

### Practice performance indicators

Practice performance indicators were chosen from publicly available datasets to reflect both incentivised and non-incentivised measures of clinical activity, and patient reported indicators. Table 2 provides a detailed description of the indicators used and their units of measurement.

**Table 2:** Definitions of GP practice performance indicators used in this study

Indicator	Question [Question No.] or Indicator Code	Indicator metric
Patient reported indicators		
Access	Generally, how easy is it to get through to someone at your GP practice on the phone? [Q1]	% of patients who find it easy to get through to someone at the GP practice on the phone (Q1)
Continuity of care	How often do you see or speak to your preferred GP when you would like to? [Q8]	% of patients who see or speak to their preferred GP always, almost always or a lot of the time (Q8)
Confidence and trust	During your last general practice appointment, did you have confidence and trust in the healthcare professional you saw or spoke to? [Q30]	% of patients who have confidence and trust in the health care professional they see (Q30)
Patient experience	Overall, how would you describe your experience of making an appointment? [Q21]	% of patients who have good experience of making an appointment (Q21)
	Overall, how would you describe your experience of your GP practice? [Q32]	% of patients who have a good overall GP practice experience (Q32)
Satisfaction	How satisfied are you with the general practice appointment times that are available to you? [Q6]	% of patients who are satisfied with general practice appointment times available to them (Q6)
	Were you satisfied with the type of appointment (or appointments) you were offered? [Q16]	% of patients who were satisfied with the type of appointment(s) they were offered (Q16)
QOF indicators (Clinical and Public Health)		
Blood pressure control	Merger (HYP003 & HYP007)	HYP003 _ The percentage of patients aged 79 years or under with hypertension in whom the last blood pressure reading (measured in the preceding 12 months) is 140/90 mmHg or less
		HYP007 _ The percentage of patients aged 80 years or over with hypertension in whom the last blood pressure reading (measured in the preceding 12 months) is 150/90 mmHg or less
Diabetes mellitus control (HbA1C)	Merger (DM020 & DM021)	DM020 _ The percentage of patients with diabetes, on the register, without moderate or severe frailty in whom the last IFCC-HbA1c is 58 mmol/mol or less in the preceding 12 months
		DM021 _ The percentage of patients with diabetes, on the register, without moderate or severe frailty in whom the last IFCC-HbA1c is 75 mmol/mol or less in the preceding 12 months
Asthma reviews	AST007	The percentage of patients with asthma, on the register, who have had an asthma review in the preceding 12 months that includes an assessment of asthma control using a validated asthma control questionnaire, a recording of the number of exacerbations, an assessment of inhaler technique and a written personalised asthma plan
COPD reviews	COPD010	The percentage of patients with COPD, on the register, who have had a review in the preceding 12 months which included a record of the number of exacerbations and an assessment of breathlessness using the Medical Research Council dyspnoea scale
Depression reviews	DEP003	The percentage of patients aged 18 or over with a new diagnosis of depression in the preceding 1 April to 31 March who have been reviewed not earlier than 10 days after and not later than 56 days after the date of diagnosis
Cervical cancer screening	Merger (CS005 & CS006)	CS005 _ The percentage of women eligible for screening and aged 25–49 years at the end of the reporting period whose notes record that an adequate cervical screening test has been performed in the preceding 3 years and 6 months
		CS006 _ The percentage of women eligible for screening and aged 50–64 years at the end of the reporting period whose notes record that an adequate cervical screening test has been performed in the preceding 5 years and 6 months
Immunisation rate	Merger (VI001, VI002, VI003 & VI004)	VI001 _ The percentage of babies who reached 8 months old in the preceding 12 months, who have received at least 3 doses of a diphtheria, tetanus and pertussis containing vaccine before the age of 8 months
		VI002 _ The percentage of children who reached 18 months old in the preceding 12 months, who have received at least 1 dose of MMR between the ages of 12 and 18 months.
		VI003 _ The percentage of children who reached 5 years old in the preceding 12 months, who have received a reinforcing dose of DTaP/IPV and at least 2 doses of MMR between the ages of 1 and 5 years
		VI004 _ The percentage of patients who reached 80 years old in the preceding 12 months, who have received a shingles vaccine between the ages of 70 and 79 years
Non-QOF clinical indicators		
Antibiotic prescription rate (crude measure)	Merger of data for Q1, Q2 & Q3 of the year 2022	Total number of antibiotic items prescribed in primary care in a general practice per 1000 registered patients per quarter. A lower value is associated with fewer items of antibiotics being prescribed, a proxy indicator of good antimicrobial stewardship.
Cancer detection rate (via two-week wait referral route)^‡^	2021/2022 data (1 year)	% of all cancer cases treated in a year resulting from a Two-Week-Wait referral (calculated by dividing the number of new cancer cases treated in a year who were diagnosed via the Two Week Wait referral route by the total number of patients registered at the practice who have a date of first treatment in the financial year on the Cancer Waiting Times system).
Cancer emergency presentation^†^	2021/2022 data (1 year)	The crude rate of persons diagnosed with cancer via an emergency route (hospital A&E) divided by the number of persons in the practice list, expressed as a rate per 100,000 persons

‡Cancer diagnosis via urgent two−week−wait referral means that GPs can recognise cancer symptoms when they present and immediately refer patients for appropriate investigations and eventual diagnosis

†Diagnosis via presentation at the Accident and Emergency (A&E) Department is a poor indicator because it entails that the symptoms were missed at the GP (in England the first point of contact between a patient and the health care system is the GP practice). So, A&E presentation means that symptoms may have gone unrecognised for a long time, missing the opportunity to catch cancer early, when treatment is most effective

### Measuring practice performance according to LPA grouping

Linear regression was used to explore the association between the derived practice groups and the selected primary care performance indicators. To mitigate the effect of uncertainty in class assignment the regression model was weighted by the probabilities of group membership. This ensured that the contribution of each practice to their assigned group was only as much as their probability of being in that group. First, unadjusted linear regression was performed between practice group and practice performance. Second, a multivariable linear regression adjusting for sex, age (proportion of registered population aged ≥ 65 years), deprivation and rurality was performed to assess the independent effect of practice group on practice performance and the robustness of the effect size. All data analyses were on practice level, with practice group as the predictor variable and practice performance indicators as dependent variables in a regression model.

## Results

Six thousand and twenty-four (6024) general practices (92.7% of practices known to exist in England as of June 2022 and covering 60,156,982 registered patients) were available for LPA class assignment. We determined that a 3-class grouping provided the most meaningful interpretation. All practices had a class assignment probability ≥ 50%, with more than 85% of practices in each group having been assigned with a probability of at least 80% (Table 3).

**Table 3:** Descriptive characteristics of the 3-Class solution

Class/group	Class size (*n*, %)	Minimum probability for class membership	% of practices with class membership probability > = 0.8
1	4494 (74.6)	0.5001	95.4
2	1400 (23. 2)	0.5037	85.3
3	131 (2.2)	0.5434	97.7

Entropy = 0.92; Bootstrap Likelihood Ration Test (BLRT) p−value = 0.01

### Characteristics of derived practice groups

Group 1 practices were characterised by a relatively small list size and a workforce that was predominantly GPs, labelled ‘Small practices more reliant on GPs’. Group 2 practices were characterised by a medium-size patient list and a multidisciplinary workforce which was dominated by GPs, labelled ‘Medium-size GP-led practices with a multidisciplinary team (MDT) input’. The third group was characterised by a large patient list and a more multidisciplinary team. GPs were still dominating, but other types of practitioners were present in significant numbers implying they had fully embraced multidisciplinary working. We labelled this group ‘Large multidisciplinary practices’. The p-values for comparing the three groups were significantly different on all indicator variables as well as on other practice characteristics (Table 4).

**Table 4:** Characteristics of GP practices across the three groups

Variable		Mean (SD, FTE/10k patients)‡			p-value†
		Small practices reliant on GPs	Medium-size, GP-led practices with MDT input	Large multidisciplinary practices	
LPA variables	Practice list size	7432 (3217)	15,775 (4686)	35,709 (14,913)	< 0.001
	General Practitioner (GP)	4.08 (2.52, 5.49)	9.67 (3.53, 6.13)	18.65 (8.35, 5.22)	< 0.001
	Nurse specialist	0.41 (0.66, 0.55)	1.43 (1.48, 0.91)	3.94 (3.06, 1.10)	< 0.001
	Nurse generalist	1.29 (0.86, 1.74)	3.37 (1.46, 2.14)	8.76 (4.16, 2.45)	< 0.001
	Pharmacist	0.46 (1.03, 0.62)	1.38 (2.12, 0.87)	3.25 (3.68, 0.91)	< 0.001
	Allied Healthcare Professional (AHP)	0.13 (0.42, 0.17)	0.59 (1.01, 0.37)	2.17 (2.18, 0.61)	< 0.001
	Health Care Assistant (HCA)	0.77 (0.66, 1.04)	2.12 (1.16, 1.34)	5.49 (2.95, 1.54)	< 0.001
	Administration	6.58 (2.85, 8.85)	14.73 (4.51, 9.34)	33.15 (13.6, 9.28)	< 0.001
Other practice characteristics	Female -to-Male ratio	0.98 (0.09)	1.01 (0.06)	1.00 (0.07)	< 0.001
	% of practice population ≥ 65	17.60 (7.25)	19.40 (6.73)	18.6 (7.29)	< 0.001
	Deprivation score	23.9 (11.9)	21.0 (10.3)	21.4 (9.20)	< 0.001
	Rurality (n, %)				
	Rural	800 (18.03)	209 (15.08)	20 (15.38)	0.034
	Urban	3638 (81.97)	1177 (84.92)	110 (84.62)	
	Contract type (n, %)				
	General Medical Services (GMS)	3270 (72.79)	991 (70.79)	93 (70.99)	< 0.001
	Personal Medical Services (PMS)	1064 (23.69)	393 (28.07)	34 (25.95)	
	Alternative Provider Medical Services (APMS)	158 (3.52)	16 (1.14)	4 (3.05)	

†Statistical testing by One−way Analysis of Variance (One−way ANOVA) for continuous variables and Pearson Chi square for categorical variables

‡Average full−time equivalents expressed per 10,000 patients (FTE/10k) only apply to workforce variables

### Practice performance by group

Performance was higher in small GP reliant practices on most patient reported indicators. The average proportion of patients who reported finding it easy to get through to someone at their GP practice on the phone was 20.4% lower (48.5% vs. 60.9%) for medium-size practices with MDT input and 36% lower (39.0% vs. 60.9%) for large multidisciplinary practices compared to small GP reliant practices (*p* < 0.001). Similarly, continuity of care was 20.9% poorer (32.6% vs. 41.2%) for medium size practices with MDT input and 34.5% worse (27.0% vs. 41.2%) for large multidisciplinary practices compared to small GP dependent practices (*p* < 0.001). The pattern is the same for all other patient reported indicators (Table 5).

The three groups also exhibited significant differences on some QOF incentivised clinical indicators (asthma reviews, cervical cancer screening and immunisation coverage) but no significant differences on others (blood pressure control, diabetes control, COPD reviews and depression reviews) in univariate (ANOVA) assessments. There were, however, significant differences between the three groups on all non-incentivised clinical indicators (antibiotic prescription rate, cancer detection rate and emergency cancer presentations) (Table 5).

**Table 5:** Patterns of practice performance across the three groups

Indicator	Indicator description	Mean percentage (SD)			*p*-value†
		Small practices reliant on GPs	Medium-size, GP-led practices with MDT input	Large multidisciplinary practices	
Access	% of patients who found it easy to get through to someone at the GP practice on the phone (Q1)	60.9 (22.0)	48.5 (20.1)	39.0 (19.4)	< 0.001
Continuity of care	% of patients who saw or spoke to their preferred GP always, almost always or a lot of the time (Q8)	41.2 (17.6)	32.6 (15.6)	27.0 (15.2)	< 0.001
Confidence and trust	% of patients who had confidence and trust in the health care professional they saw (Q30)	92.6 (5.4)	93.7 (4.3)	93.2 (4.2)	< 0.001
Patient experience	% of patients who had good experience of making an appointment (Q21)	60.0 (16.5)	55.2 (14.7)	49.8 (13.7)	< 0.001
	% of patients who had a good overall GP practice experience (Q32)	74.1 (13.4)	72.7 (11.9)	68.1 (11.4)	< 0.001
Satisfaction	% of patients who were satisfied with general practice appointment times available to them (Q6)	58.2 (14.7)	53.9 (13.1)	49.3 (12.8)	< 0.001
	% of patients who were satisfied with the type of appointment(s) they were offered (Q16)	72.7 (11.6)	72.1 (10.0)	69.2 (9.4)	< 0.001
Blood pressure control	% of patients who achieved blood pressure control	64.7 (12.3)	64.4 (10.6)	64.2 (10.2)	0.656
Diabetes control (HbA1C)	% of patients who had controlled diabetes control	61.8 (9.9)	62.3 (8.9)	61.6 (7.6)	0.149
Asthma reviews	% of asthmatic patients who had an adequate asthma review in the 12 months preceding data abstraction	61.8 (21.7)	59.8 (19.8)	58.2 (19.5)	0.003
COPD reviews	% of patients with COPD who had been adequately reviewed in the 12 months preceding data abstraction	66.8 (21.1)	67.3 (21.8)	65.5 (21.1)	0.649
Depression reviews	% of newly diagnosed depression patients who had a timely follow up review (≥ 10 & ≤56 days after diagnosis)	68.4 (25.8)	69.4 (23.6)	71.1 (22.8)	0.242
Cervical cancer screening	% of eligible women screened for cervical cancer	79.3 (8.37)	80.3 (7.2)	79.5 (8.72)	< 0.001
Immunisation rate	% of the population eligible for a vaccine who were vaccinated	82.9 (11.8)	85.2 (8.6)	84.1 (7.1)	< 0.001
Antibiotic prescription rate (crude)	Number of antibiotic items prescribed per 1000 patients (period = first 9 months of the year 2022)	111.0 (34.6)	114.0 (28.7)	116.0 (75.6)	0.003
Cancer detection rate	% of all cancer cases treated in 1 year diagnosed via Two-Week-Wait referral route	53.1 (12.6)	54.7 (8.5)	55.6 (7.1)	< 0.001
Emergency cancer presentation	Crude rate of persons diagnosed with cancer via an emergency route, expressed per 100,000 persons	89.1(50.8)	94.7 (38.5)	91.6 (41.8)	0.001

†Statistical testing by One Way Analysis of variance (one way ANOVA)

### Association between practice group and performance

Compared to small GP-reliant practices, larger practices with multidisciplinary teams performed poorly in patient reported indicators (Table 6). Medium-size practices with MDT input and large multidisciplinary practices had on average 13.5% and 22.8% fewer patients respectively who reported finding it easy getting through to someone on the phone at their GP practice compared to patients in small GP-reliant practices. Continuity of care fell by 9.3% points on average in medium-size practices with MDT input and 15.1% points on average in large multidisciplinary practices compared to small GP-led practices. Patient experience of making a GP appointment was, on average, 5.8% points lower in medium-size practices with MDT input and 11.1% points lower in large multidisciplinary practices compared to small GP-reliant practices. Good overall experience with the general practice was 2.7% points lower in medium-size practices with MDT input and 7.0% points lower in large multidisciplinary practices on average compared to small GP-reliant practices. Satisfaction with appointment times was on average 4.7% points lower in medium-size practices with MDT input and 9.2% points lower in large multidisciplinary practices compared to small GP-reliant practices. When it came to confidence and trust, the percentage of patients who reported having confidence and trust in the healthcare professional they saw on their last GP appointment was on average 0.3 points higher in medium-size practices with MDT input compared to small GP-reliant practices. No difference was observed between large multidisciplinary practices and small GP-reliant practices.

**Table 6:** Regression coefficients for the association between practice group and practice performance indicators

Indicator^†^	Indicator description	Unadjusted regression coefficient (95% CI)			Adjusted regression coefficient (95% CI)^‡^		
		Small practices reliant on GPs (ref group)	Medium-size, GP-led practices with MDT input	Large multidisciplinary practices	Small practices reliant on GPs (ref group)	Medium-size, GP-led practices with MDT input	Large multidisciplinary practices
Access	Getting through to someone at the GP practice on the phone (Q1)	0	-12.88 (-14.19 – -11.58)***	-22.18 (-25.87 – -18.49)***	0	-13.51 (-14.79 – -12.23)***	-22.75 (-26.29 – -19.22)***
Continuity of care	Seeing or speaking to preferred GP (Q8)	0	-8.86 (-9.90 – -7.81)***	-14.43 (-17.38 – -11.48)***	0	-9.26 (-10.29 – -8.22)***	-15.06 (-17.93 – -12.18)***
Confidence and trust	Confidence and trust in health care professional (Q30)	0	1.05 (0.74 – 1.37)***	0.55 (-0.34 – 1.44)	0	0.33 (0.04 – 0.62) *	0.03 (-0.78 – 0.83)
Patient experience	Experience of making appointment (Q21)	0	-5.02 (-6.00 – -4.05)***	-10.39 (13.14 – -7.63)***	0	-5.80 (-6.75 – -4.85)***	-11.09 (-13.72 – -8.45)***
	Overall GP practice experience (Q32)	0	-1.46 (-2.26 – -0.67)***	-6.03 (-8.27 – -3.79)***		-2.74 (-3.49 – -1.98)***	-6.99 (-9.08 – -4.89) ***
Satisfaction	Satisfaction with general practice appointment times available to them (Q6)	0	-4.51 (-5.37– -3.63)***	-8.98 (-11.44 – -6.52)***	0	-4.73 (-5.59 – -3.86) ***	-9.24 (-11.65 – -6.83)***
	Satisfaction with the type of appointment(s) offered to them (Q16)	0	-0.64 (-1.33 – 0.04)	-3.56 (-5.49 – -1.63)***	0	-1.45 (-2.11 – -0.79)***	-4.21 (-6.02 – -2.39 )***
Blood pressure control	Percentage achieved blood pressure control	0	-0.36 (-1.08 – 0.37	-0.57 (-2.61 – 1.48)	0	-0.28 (-1.02 – 0.46)	-0.44 (-2.49 – 1.61)
Diabetes control (HbA1C)	Percentage achieved diabetes control	0	0.52 (-0.07 – 1.11)	-0.20 (-1.87 – 1.46)	0	-0.04 (-0.63 – 0.55)	-0.56 (-2.19 – 1.07)
Asthma reviews	Percentage of asthmatic patients who had an adequate asthma review in the 12 months preceding data retrieval	0	-2.14 (-3.43 – -0.85)**	-3.72 (-7.36 – -0.07)*	0	-1.51 (-2.83 – -0.19)*	-3.33(-6.98 – 0.32)
COPD reviews	Percentage of patients with COPD who had been adequately reviewed in the 12 months preceding data abstraction	0	0.52 (-1.00–2.03)	-1.30 (-5.60–3.00)	0	0.79 (-0.76 – 2.34)	-1.03 (-5.35 – 3.29)
Depression reviews	Percentage of newly diagnosed depression patients who had a timely follow up review (≥ 10 & ≤56 days after diagnosis)	0	0.94 (-0.59 – 2.48)	2.59 (-1.75 – 6.92)	0	0.45 (-1.12 – 2.02)	2.47 (-1.87 – 6.81)
Cervical cancer screening	Percentage of eligible women screened for cervical cancer	0	0.92 (0.43 – 1.42)***	0.12 (-1.27 – 1.52)	0	0.03 (-0.44 – 0.50)	-0.24 (-1.54 – 1.07)
Immunisation rate	Percentage of the population eligible for a vaccine who were vaccinated	0	2.29 (1.62 – 2.97)***	1.14 (-0.76 – 3.05)	0	1.17 (0.52 – 1.83)***	0.41 (-1.40 – 2.22)
Antibiotic prescription rate (crude)	Number of antibiotic items prescribed per 1000 patients (period = first 9 months of the year 2022)	0	3.36 (1.24 – 5.49)**	4.70 (-1.29 – 10.69)	0	-0.18 (-1.89 – 1.54)	3.81 (-0.95 – 8.57)
Cancer detection rate	% of all cancer cases treated in 1 year diagnosed via a Two-Week-Wait referral route	0	1.72 (1.00 – 2.43)***	2.58 (0.56 – 4.60)*	0	0.92 (0.22 – 1.63)*	2.03 (0.07 – 3.99)*
Emergency cancer presentation	Crude rate of persons diagnosed with cancer via an emergency route per 100,000 persons	0	5.69 (2.76 – 8.62)***	2.61 (-5.68 – 10.89)	0	0.33 (-2.14 – 2.81)	0.37 (-6.49 – 7.24)

Statistical significance: *** *p* < 0.001; ** *p* < 0.01; * *p* < 0.05

‡Adjusted for percentage of patients ≥65 years, female−to−male sex ratio, deprivation and rurality

†Practice performance indicators are practice level percentages except antibiotic prescription and emergency cancer presentation which are rates

For QOF incentivised clinical indicators, medium-size practices with MDT input attained fewer QOF points related to asthma reviews (1.5 points less on average) but achieved higher immunisation rate (1.2 points higher on average) compared to small GP-reliant practices. In terms of non-QOF clinical indicators, larger multidisciplinary practices performed better at detecting cancer early. Medium-size practices with MDT input had, on average, 0.9 points increase while large multidisciplinary practices had 2.0 points increase in the proportion of cancers diagnosed via the two-week-wait referral route. The three groups exhibited no significant difference on other clinical indicators assessed (Table 6).

## Discussion

### Summary of main findings

General practices in England can be classified into three groups: (1) Small and GP-reliant (2) Medium size with a multidisciplinary team (MDT) input, and (3) Large and multidisciplinary. The majority (75%) of practices in England are small and reliant on GPs.

Large and medium-size practices performed worse on all patient reported indicators except confidence and trust in healthcare professionals where although medium-size practices with MDT input appeared to do better than small GP-reliant practices, the effect size was small.

Groups performed similarly for incentivised clinical indicators. Medium-size practices performed better on immunisation coverage and worse in asthma reviews compared to small practices, but the effect sizes were small and did not extend to large multidisciplinary practices as one would expect.

Considering non-incentivised clinical indicators, larger practices with multidisciplinary teams appeared to do better at catching cancer early compared to small GP-reliant practices as measured by the proportion of cancer cases treated that were diagnosed via the two-week-wait pathway.

### Strengths

This is the first study in England to have used finite mixture modelling to group practices into different organisational models based on list size and workforce composition, and assess the effect of these different organisational models on practice performance. It represents a departure from previous research where practice size was defined in terms absolute list size (number of patients registered at a practice), list size per GP or as single-handed (owned by 1 GP) versus multiple-handed (multiple GP partners) practices.

We assessed practice performance in diverse outcomes, ranging from patient reported to clinical and preventive care indicators (both incentivised and non-incentivised).

### Limitations

There was some uncertainty in class membership, especially for the medium-size practices with MDT input group which had nearly 15% of practices assigned to it with probability < 0.8. This was mitigated by weighting the regression analysis by class membership probabilities.

Most dependent variables were practice level percentages, bound between 0 and 100. It is difficult to appropriately fit linear models with bounded variables. This can lead to predictions that are outside the plausible range (negative or above 100) or generate coefficients that are higher or lower than the actual mean differences between the groups [27, 28]. Fortunately, we did not observe any out-of-range predictions.

In the 2022 General Practice Patient Survey, only 29% of targeted participants responded. Such a low response rate raises questions about the representativeness of the sample. This problem is, however, mitigated by the fact that the GPPS results are weighted to account for selection bias and differences in demographic characteristics between responders and non-responders [29].

We did not control for other confounders in primary care such as prevalence of chronic diseases, patient turnover and proportion of patients born in a developing country. Nonetheless, previous research demonstrated that these have no effect on clinical outcomes as measured by practice QOF points [30].

Capturing practice level workforce composition is complicated by a number of roles that are employed at PCN level as specified in the Additional Roles Reimbursement Scheme (ARRS) [31]. Staff employed at PCN would not be reported as practice employees in the datasets used in this study, despite working in practices and contributing significantly to the pattern of the workforce. The ARRS roles make-up a significant proportion of the non-GP workforce and future research would be strengthened by inclusion of this data.

Workforce data include staff on long-term absence due to sickness, maternity/paternity leave among other reasons and temporary staff that are recruited to cover for these absences which inflates the Figures [32]. Furthermore, Workforce data presents a snapshot of GP practice workforce [32]. They do not tell us how long different staff roles have been available in the practice to make any meaningful impact. Patient list sizes, on the other hand, are inflated by practices’ delay or failure to deregister patients who have left the practice [33].

This study used GP datasets for England, which is actively encouraging their GP practices to work at scale and have multidisciplinary teams. Therefore, the results may not apply to the rest of the United Kingdom or other countries. Nonetheless, these results provide caution to countries pursuing or considering similar policies.

### Results in relation to other studies

Similar to our findings, previous studies have generally reported that smaller practices outperformed larger practices on patient reported indicators irrespective of how practice size was defined [11, 13, 34–37].

For clinical outcomes, previous research favours larger practices. Group practices achieved higher QOF points than single-handed practices [30]. Larger practices also had better diabetes control [10, 11, 35], vaccination rates [38], cancer screening [39], depression reviews [40], antibiotic use [41], specialist referrals [42] and use of clinical guidelines [43] than smaller practices. No differences were found between smaller and larger practices on blood pressure and cholesterol control [44], use of diagnostic investigations [40, 45, 46] or medication prescription [40, 44–46]. We did not find compelling evidence for better clinical outcomes in larger multidisciplinary practices except that larger multidisciplinary practices appeared to do better at recognising cancer symptoms earlier and referring patients to specialists sooner. This discrepancy may be because most of the quality indicators we used are financially incentivised in England.

Continuity of care has been associated with better clinical outcomes, especially in chronic diseases such as hypertension [47] and diabets [48]. It is also associated with fewer emegency room attendances [48, 49], fewer hospitalisations [12, 48], high uptake of immunisations [50] and low mortality [48, 51]. It is belived that this is is the case because continuity leads to doctors accumulating more knowledge about their patients and their condition, and develop a sense of responsility towards them which in turn leads to more personalised care [52]. This was not reflected in our study. We believe this has to do with how continuity has been conceptualised. Traditionally, continuity has been defined as repeated contacts with the same doctor over time [53]. Consequently, in the GPPS, respondents were asked how often they saw their preferred GP. But chronic disease care is often provided by a multidisciplinary team of practitioners including nurses and pharmacists, and relationships are built with teams not individuals. Perhaps an alternative definition of continuity that includes nurses and AHPs might capture the relationships built with other practitioners and better reflect the impact on clinical outcomes.

### Implications for practice and future research

The lack of significant differences found in clinical outcomes between large multidisciplinary practices, medium-size practices with MDT input and small GP-reliant practices may be a reflection of the fact that larger multidisciplinary practice models are relatively new and yet to start reaping the benefits of working at scale. Longitudinal studies to assess whether changes in practice’s organisational structure over time produce incremental gains in key indicators would be beneficial. Further, we only included practice-level workforce data, future studies with PCN-level data [54] are needed.

There is need to expedite efforts to accurately capture the activity of different staff groups in GP practice. For instance, the General Practice Appointments data currently does not provide a detailed breakdown of appointments by healthcare professional type. Appointments are categorised into just two groups: those attended by a GP and those attended by other practice staff (i.e., appointments delivered by different DPC staff are reported together) [55]. Improving documentation of the activity of these new practitioners is needed to better understand which practitioner type is making an impact in primary care. It is using the wider multidisciplinary team more effectively that has the potential to increase access and provide longer appointments, which have been associated with increased satisfaction and positive clinical outcomes elsewhere [11, 35].

In addition, more understanding of whether different practitioners are being utilised effectively is needed because as new roles evolve there is potential for challenges of integration into the existing primary care team [56]. It is important to clearly define their scope of work and need for supervision so managers can monitor and optimise the working environment for all staff.

Furthermore, to produce optimal results large multidisciplinary practices will require substantial financial and infrastructural investments (estates, medical equipment and information technology) [57].

## Conclusion

English general practices can be described as small and GP-reliant, medium-size with MDT input and large and multidisciplinary. There is evidence that patients strongly prefer smaller more GP-led practices, thanks to more accessible and personalised care they are perceived to provide. There were minimal differences in clinical outcomes between the three groups but some indication that larger multidisciplinary practices may perform better in cancer referrals. Since primary care at scale remains the current political agenda, care should be taken to ensure that as practices merge or enter collaborations the features of traditionally small, GP-led general practice that patients hold dear, and generally lead to similar clinical outcomes, are not lost.

## Data Availability

This study used publicly available data which have been duly cited in text, with links to online sources provided in the reference list.

## Abbreviations

GP: General Practitioner (a primary care doctor in England)
GP practice: General Practitioner practice (an organisation providing primary care in England)
PCN: Primary Care Network
NHS: National Health Service
LPA: Latent Profile analysis
QOF: Quality and Outcomes Framework
GPPS: General practice patient survey
IMD: Index of Multiple Deprivation
MDT: Multidisciplinary team
DPC: Direct Patient Care (non-GP staff that provide direct patient care)
ARRS: Additional Roles Reimbursement Scheme
COPD: Chronic Obstructive Pulmonary Disease
